# A novel approach to designing viral precision vaccines applied to SARS-CoV-2

**DOI:** 10.3389/fcimb.2024.1346349

**Published:** 2024-04-02

**Authors:** Khaled Trabelsi, Noureddin Ben Khalaf, Ahmed R. Ramadan, Amany Elsharkawy, Dana Ashoor, Sadok Chlif, Thouraya Boussoffara, Melika Ben-Ahmed, Mukesh Kumar, M-Dahmani Fathallah

**Affiliations:** ^1^ Health Biotechnology Program, King Fahad Chair for Health Biotechnology, Department of Life Sciences College of Graduate Studies, Arabian Gulf University, Manama, Bahrain; ^2^ Department of Biology, College of Arts and Sciences, Georgia State University, Atlanta, GA, United States; ^3^ Department of Family and Community Medicine, College of Medicine and Medical Sciences, Arabian Gulf University, Manama, Bahrain; ^4^ Transmission, Control and Immunobiology of Infections Laboratory, Institute Pasteur of Tunis, Tunis, Tunisia

**Keywords:** vaccine, precision, SARS-CoV-2, antigen (Ag), infectivity

## Abstract

Efficient precision vaccines against several highly pathogenic zoonotic viruses are currently lacking. Proteolytic activation is instrumental for a number of these viruses to gain host-cell entry and develop infectivity. For SARS-CoV-2, this process is enhanced by the insertion of a furin cleavage site at the junction of the spike protein S1/S2 subunits upstream of the metalloprotease TMPRSS2 common proteolytic site. Here, we describe a new approach based on specific epitopes selection from the region involved in proteolytic activation and infectivity for the engineering of precision candidate vaccinating antigens. This approach was developed through its application to the design of SARS-CoV-2 cross-variant candidates vaccinating antigens. It includes an *in silico* structural analysis of the viral region involved in infectivity, the identification of conserved immunogenic epitopes and the selection of those eliciting specific immune responses in infected people. The following step consists of engineering vaccinating antigens that carry the selected epitopes and mimic their 3D native structure. Using this approach, we demonstrated through a Covid-19 patient-centered study of a 500 patients’ cohort, that the epitopes selected from SARS-CoV-2 protein S1/S2 junction elicited a neutralizing antibody response significantly associated with mild and asymptomatic COVID-19 (p<0.001), which strongly suggests protective immunity. Engineered antigens containing the SARS-CoV-2 selected epitopes and mimicking the native epitopes 3D structure generated neutralizing antibody response in mice. Our data show the potential of this combined computational and experimental approach for designing precision vaccines against viruses whose pathogenicity is contingent upon proteolytic activation.

## 1 Introduction

While vaccines developed to combat severe acute respiratory syndrome coronavirus 2 (SARS-CoV-2) helped mitigate the coronavirus disease 2019 (COVID-19) pandemic (Angeli et al., 2021; Gibson et al., 2023), numerous highly pathogenic zoonotic viruses, such as Ebola, Zika, Dengue, West Nile, MERS-CoV and a few others that can potentially cause pandemics, still lack efficient vaccines (Maslow, 2017; Reperant and Osterhaus, 2017; Trovato et al., 2020). As for SARS-CoV-2, the continual emergence of more transmissible genetic variants that escape the immune response (Krause et al., 2021) to previous variants is raising concerns about the resurgence of the COVID-19 pandemic. The SARS-CoV-2 currently available vaccines use a conventional approach to choosing vaccinating antigens. Indeed, these vaccines are based on a non-precision targeting approach using the entire S protein as an antigen (Walls et al., 2020b). Careful selection of vaccinating antigens is important as some SARS-CoV-2 epitopes have been shown to generate deleterious immune response such as antibody-dependent enhancement (ADE) of the infection clinical course (Karthik et al., 2020; Lee et al., 2020). ADE is generally secondary to the immune response to cross-reactive antigens encountered in previous exposure to coronavirus strains (Fierz and Walz, 2020), or H3N2 Influenza A virus (Sen et al., 2021). Therefore, a new design concept is needed to develop efficient precision vaccines against SARS-CoV-2 and other concerning viruses, particularly those prone to genetic variations. The current COVID-19 vaccines elicit a good immune response, namely, a humoral response with neutralizing antibodies (Long et al., 2020; Okba et al., 2020; Zhao et al., 2020; Earle et al., 2021). Nevertheless, these vaccines showed variable efficacy, from very high to moderate, and none conferred sterilizing immunity. Furthermore, some instances of concerning undesirable effects, such as thrombocytopenia and micro blood clots, cardiac tissue inflammation and even possible reprogramming of the immune response, have been reported (Trogen et al., 2020; Föhse et al., 2021; Menni et al., 2021; Thompson et al., 2021). An approach based on the identification of epitopes eliciting an immunity that interferes precisely with viral cell entry and controls infectivity could be very useful for the design of precision vaccines. This is particularly relevant because for most pathogenic human viruses, the structure responsible for cell entry and infectivity undergoes precisely orchestrated specific proteolysis carried out by numerous proprotein convertases (Babe and Craik, 1997), with furin being the most reactive (Izaguirre, 2019). Immunity that hampers this process can control viral infectivity.

Compared to common coronaviruses, SARS-CoV-2 has higher infectivity and enhanced pathogenicity, which allowed it to cause the COVID-1 9 pandemics. In this respect, comparison of the structural features of SARS-CoV-2 with those of other coronaviruses revealed differences that account for the enhanced infectivity. These differences particularly relate to the coronavirus spike (S) protein (Walls et al., 2020a), which plays a key role in the early steps of viral infection. One particularly striking feature of the SARS-CoV-2 genome revealed by comparative genomic studies is the presence of a polybasic furin cleavage site (FCS) at the S1/S2 boundary after the insertion of 12 nucleotides encoding the four amino acid residues PRRA downstream of the transmembrane protease serine 2 (TMPRSS2) S’2 cleavage site. This FCS is not present in the S proteins of any of the less pathogenic coronaviruses of the same clade (Coutard et al., 2020). Furin cleavage strongly increases cleavage at the S1/S2 boundary (Vankadari, 2020). This proteolytic cleavage of the SARS-CoV-2 S protein acts in concert with cleavage by the host cell protease TMPRSS2 at the S2’ site downstream of S2 (Fuentes-Prior, 2021; Jackson et al., 2022). This double proteolytic processing of the S protein by furin and TMPRSS2 liberates the fusion peptide (FP) located immediately downstream of the TMPRSS2 site and allows virus-host cell membrane fusion and virus cell entry. This is probably a major determinant of the high infectivity of SARS-CoV-2 (Lavie et al., 2022). The presence of the FCS has been associated with SARS-CoV-2 pathogenesis, which is consistent with the high infectivity of SARS-CoV-2 (Johnson et al., 2021).

Therefore, identifying immunogenic epitopes within this region can help develop a vaccine likely to generate an immune response that restricts the activation of the virus and thus mitigates infectivity. Most individuals infected with SARS-CoV-2 develop an antibody response following infection (Long et al., 2020; Okba et al., 2020; Zhao et al., 2020). The detection of IgG antibodies is important for tracking immunity to SARS-CoV-2. Indeed, IgG antibodies last for several months (Yousefi et al., 2022) and, along with IgA antibodies, are generally endowed with antiviral activity. Antibodies that have antiviral activity are generally associated with protective immunity and recovery from COVID-19 (Earle et al., 2021; Khoury et al., 2021; Focosi et al., 2022). The presence of antibodies directed to epitopes in the S protein region involved in SARS-CoV-2 activation and enhanced infectivity has not been fully investigated.

In this paper, we describe a new approach to design an antiviral precision candidate vaccinating antigens for highly pathogenic viruses through its application to the design of SARS-CoV-2 vaccine antigens. This approach is based on targeting predicted epitopes in the virus structural region involved in activation and infectivity. We have engineered antigens candidates that structurally mimic predicted epitopes in such region of SARS-C0V-2. These antigens generated a strong and long-lasting antibody response in mice. We also showed that the sera of non-vaccinated patients with COVID-19, recognize these antigens and that this antibody response correlates with natural immune protection. This approach offers a rational way to design viral precision vaccines that interfere with infectivity and likely reduce pathogenicity.

## 2 Materials and methods

### 2.1 Patient selection and serum preparation

A cohort of 500 COVID-19 non-vaccinated patient volunteers were obtained from Asry Medical Center and Awali Hospital in Manama, Bahrain. The selection was based on a positive SARS-CoV-2 PCR test, age range between 18 and 75 years and a clear clinical status defined by the patients’ attending physicians according to World Health Organization (WHO) COVID-19 classification as asymptomatic, mild, moderate and severe forms (https://www.who.int/westernpacific/emergencies/covid-19/information/asymptomatic-covid-19. Information on this cohort is compiled in **Table 1**. The patient’s sera were prepared and stored at -80°C. One hundred pre-pandemic historical sera from age-matched healthy individuals were also retrieved and used as negative controls for this study. Informed consent was obtained from all patients and control individuals, and approval was obtained from the Arabian Gulf University Institutional Research and Ethics Committee on December 24, 2020, reference E031-PI-12/20. All methods were performed in accordance with the principles of Helsinki declaration for Medical Research Involving Human Subjects.

**Table 1:** SARS-CoV-2-infected patient and healthy donor cohort characteristics.

		HEALTHY DONORS*	COVID-19 PATIENTS*
AGE [years]			
Average		40.7	43.0
Range		[21-66]	[21-68]
SEX			
Male		69	470
Female		31	30
PCR POSITIVITY		NA	500
Range of sampling time from date of positive PCR testing		NA	2.5 to 55 weeks
DISEASE STATUS	Code		
Asymptomatic	0	NA	310
Mild	1	NA	124
Moderate	2	NA	29
Severe/critical	3	NA	37
SAMPLE COLLECTION PERIOD		January–May 2021	

*All the patients and healthy donors were not vaccinated at the time of DNA sampling.

### 2.2 Animals

Purpose-bred BALB/c mice used in this study were housed in stainless steel ventilated cages under a 12-hour light/12-hour dark cycle at room temperature ranging from 18 to 22°C and a humidity between 50 and 60%. Animals were fed rodent chow and water ad libitum. Experiments were conducted according to national and international guidelines, such as those issued by the US National Institute of Health Office of Laboratory Animal Welfare (OLAW) (https://olaw.nih.gov/home.html) the study is reported in accordance with ARRIVE guidelines (https://arriveguidelines.org). The university Research and Ethics committee approved the protocols (reference E031-PI-12/20).

### 2.3 Retrieval of the S protein monomer structure and analysis of the SARS-CoV-2 S protein S1/S2 junction sequence surface

The coordinates of the SARS-CoV-2 S protein trimeric form (PDB ID: 6VXX) were downloaded from the RCSB database (https://www.rcsb.org). Water and heteroatoms were removed from the coordinates, and the coordinates of the complex were split into separate PDB monomer files using PyMOL software (Delano, 2002) from the Schrodinger software package R. Because the PDB ID: 6VXX model is missing the furin and TMPRSS2 cleavage site loops, the S protein monomer was modelled to include these loops using the Phyre2 web server (http://www.sbg.bio.ic.ac.uk/phyre2/html/page.cgi?id=index) (Kelley et al., 2015), and the results were saved on PyMOL software to visualize exposed residues.

### 2.4 B-cell epitope prediction

The S protein amino acid sequence was submitted to the BepiPred-2.0 Sequential B-Cell Epitope Predictor server (http://www.cbs.dtu.dk/services/BepiPred/cite.php) using the default parameters to predict putative immunogenic epitopes in the S protein monomer surface-exposed regions. BepiPred-2.0 is based on a random forest algorithm trained on epitopes and non-epitopes annotated from antibody-antigen protein crystal structures (Jespersen et al., 2017).

### 2.5 Prediction of proteasome and immunoproteasome cleavage sites

The S protein S1/S2 junction sequence (residues E648-L809) containing the furin and TMPRSS2 proteolysis sites was submitted to the improved Proteasome Cleavage Prediction Server (http://imed.med.ucm.es/Tools/pcps/). This server predicted the cleavage sites generated by the constitutive proteasome/immunoproteasome within the protein sequence using different Ngram models (Garcia-Boronat et al., 2008) and displayed the predicted epitopes.

### 2.6 Prediction of T-cell epitopes and binding to MHC class 1 and class 2

To predict MHC class 1-binding peptides, we used the NetMHCpan 4.1 (https://services.healthtech.dtu.dk/services/NetMHCpan-4.1/) and NetMHC - 4.0 (https://services.healthtech.dtu.dk/services/NetMHC-4.0/) servers. NetMHCpan and NetMHC servers operate on artificial neural networks (ANNs) to predict the binding of peptides to MHC class I molecules of known sequences. To predict MHC class 2 peptides, we used the NetMHCII 2.3 (https://services.healthtech.dtu.dk/services/NetMHCII-2.3/) (Jensen et al., 2018), which allows for the prediction of peptides binding to HLA-DR, HLA-DQ, and HLA-DP. We analyzed The S protein S1/S2 junction sequence using the default settings of all servers. Peptides were then sorted by the software according to their binding affinity level to MHC molecules. Binding affinity prediction methods for NetMHCII were constructed using an extended data set of quantitative MHC–peptide binding affinity data obtained from the Immune Epitope Database covering HLA-DR, HLA-DQ, HLA-DP and H-2 molecules (Jensen et al., 2018).

### 2.7 Conservation of the predicted epitopes

We used BioEdit, a multiple sequence alignment (Hall, 1999) software program, to analyze the conservation of the identified epitopes. We used the Epitope Conservancy Analysis tool from the freely accessible Immune-Epitope Database (IEDB; http://tools.iedb.org/conservancy/) to assess the variability of the epitopes based on the sequence alignment of 25 SARS-CoV-2 variants that have circulated or are still circulating since the start of the pandemic, along with SARS-CoV and MERS-CoV sequences. We acquired the amino acid sequences of the extracellular domain of SARS-CoV, MERS-CoV, and SARS-CoV-2 spike protein from the National Center for Biological Information (NCBI) protein ID: YP_009825051.1, YP_009047204.1 and YP_009724390.1, respectively. We reproduced SARS-CoV-2 variants sequences by introducing specific mutations to the Wuhan sequence to generate different variants using published mutations in the covariant (https://covariants.org/) and Stanford University SARS-CoV-2 Variants databases (https://covdb.stanford.edu/variants/omicron_ba_1_3/)

### 2.8 Prediction of IFN-γ epitopes

We used the interferon epitope server https://webs.iiitd.edu.in/raghava/ifnepitope/predict.php to identify IFN-γ-inducing epitopes in the designed polypeptide P3/Fur/x3 and protein subunits P3-L and SJ/FT.

### 2.9 3D modelling of immunogenic polypeptides and protein subunits

For *ab initio* modelling, we used Iterative Threading Assembly Refinement (ITASSER) (https://zhanglab.dcmb.med.umich.edu). All designed peptides sequences were submitted to the ITASSER server using the default settings. The server identified structural templates from the template library by using the multiple threading approach LOMETS. Functional insights regarding the target were then derived by rethreading the 3D models through the protein function database BioLiP (Yang and Zhang, 2015). The obtained models were visually inspected with PyMOL.

### 2.10 Polypeptide synthesis

The polypeptides P3/FUR/x3 and P4/TMP/x3 were chemically synthesized by GeneCust (GeneCust, Boynes, France) at a 5 mg scale and 95% minimum purity, as verified by mass spectrometry analysis and high-performance liquid chromatography (HPLC). For the polypeptides used in the indirect ELISA, a biotin tag was alternatively added to allow antigen binding to streptavidin-coated plates.

### 2.11 ELISA experiments

For the development of the indirect ELISA with the candidate vaccinating antigens, Ninety-six-well ELISA plates (Nunc MaxiSorp, Thermo Fisher Scientific) were coated with 200 ng per well of streptavidin in carbonate-bicarbonate buffer, pH 9.6 (C3041, Sigma-Aldrich), overnight at 4°C. The coated plates were then washed three times with 200 µl of wash buffer (0.1 M phosphate, 0.15 M sodium chloride, pH 7.2, 0.05% Tween 20) and blocked with wash buffer containing 5% nonfat milk “Régilait” powder at room temperature for 1 hour under shaking. Then, the plates were washed as previously described, and 100 µl of biotinylated peptides (200 ng) was added to each well. After peptide coating, the plates were washed again, 100 µl of serum sample diluted to 1:100 in dilution buffer was added to each well, and the plates were incubated at room temperature for 1 h. The plates were then extensively washed 3 times, 10000-fold-diluted horseradish peroxidase (HRP)-conjugated goat anti-human IgG (Abcam) was added, and the plates were incubated for 1 hour at room temperature. TMB substrate (TMB, Sigma-Aldrich) was added to each well, and the plates were then incubated for 20 minutes in the dark. The reaction was stopped by adding 100 μL of 2 N H2SO4 solution. The optical density (OD) was measured at 450 nm using a FLUOstar Omega (BMG LABTECH) absorbance microplate reader. We defined a sample as IgG positive if the OD value was equal to 3 standard deviations (SDs) above the mean of the negative control values (n=100).

To test the cohort of patients with COVID-19 for IgG to the SARS-CoV-2 S protein RBD region, we used a commercial quantitative ELISA kit (RayBio COVID-19 S1 RBD protein Human IgG ELISA kit; RayBiotech; Parkway, USA). The kit provides positive controls to construct a standard curve for IgG titer determination. The ELISA was performed according to manufacturer’s procedure. Briefly plates were received coated with SARS-CoV-2 S1 RBD protein, and incubated with 100µL of serum for 1h at room temperature (RT). Then, the wells are washed four times with 300µL of wash buffer, and 100µL of biotinylated anti-human IgG antibody was added and incubated for 30 minutes at RT. The wells were washed again and the TMB substrate solution was added. The reaction was stopped, and the OD was measured at 450nm.

### 2.12 Production and purification of the recombinant multiepitope protein subunits

The multiepitope subunit P3-L was designed to include a homopolymerized (3 times) 32-amino-acid core sequence containing the FCS. The SJ/FT subunit was designed to contain both the furin and TMPRSS2 cleavage motifs and the FP sequence. The two protein subunits were produced as recombinant proteins. The cDNA nucleotide sequences encoding these multiepitope subunits were chemically synthetized by GeneCust (GeneCust, Boynes, France). Each cDNA was inserted into the pET-22b (+) plasmid (Novagen Sigma-Aldrich, USA) NdeI- XhoI multiple cloning site, and the recombinant expression vector was transformed into *Escherichia coli* BL21 (DE3) cells. Positive colonies were selected on 50 µg/ml ampicillin agarose plates. For each recombinant cDNA, a single positive colony was grown overnight at 37°C on LB medium, the expression of the recombinant protein was induced by adding 0.5 mm IPTG, and bacteria were cultured at 220 rpm for 16 hours at 37°C for P3-L and at 15°C for SJ/FT. The expressed protein was purified from the sonicated bacterial pellet using a Ni-NTA column. The purified protein was dialysed in 20 mM Tris, 4 M urea, pH 8.0, at 4°C. Endotoxin was removed using endotoxin removal beads from Miltenyi Biotec (Bergischgladbach/Germany).

### 2.13 Immunogenicity and allergenicity prediction

We used the VaxiJen server (http://www.ddg-pharmfac.net/vaxijen/VaxiJen/VaxiJen.html) and ANTIGENpro module of the SCRATCH protein predictor (http://scratch.proteomics.ics.uci.edu/) to evaluate the immunogenicity of the multiepitope subunits. We used the AllerTOP v. 2.0 (http://www.ddg-pharmfac.net/AllerTOP/) and AlgPred servers (http://crdd.osdd.net/raghava/algpred/) to check for potential allergenicity.

### 2.14 Determination of physicochemical characteristics

We used the ProtParam tool of the EXPASY database server (http://web.expasy.org/protparam/) to determine the physicochemical parameters (molecular weight, half-life, atomic composition, stability index and mean hydrophilicity) of the vaccine candidates’ antigens.

### 2.15 Study of interferon-γ production by ELISpot and ELISA

Cell culture and stimulation: Peripheral blood mononuclear cells (PBMCs) were isolated from heparinized blood from 10 healthy donors and 10 patients with COVID-19 by density centrifugation through Ficoll-Hypaque (GE Healthcare Bio-Sciences AB, Uppsala, Sweden). PBMCs were cultured in 96-well plates at a concentration of 1 × 10^6^ cells/ml in a final volume of 200 µl of RPMI 1640 medium (Sigma, St. Louis, MO) supplemented with 2 mM L-glutamine (Sigma, St. Louis, MO), 100 U/ml penicillin (Sigma, St. Louis, MO), 100 µg/ml streptomycin (Sigma, St. Louis, MO), 10% (v/v) heat-inactivated human AB serum (Sigma, St. Louis, MO), 1% HEPES (0.01 M), (Invitrogen, Cergy-Pontoise, France), 1% sodium pyruvate (1 mM) (Invitrogen, Cergy-Pontoise, France), 1% MEM nonessential amino acids (Invitrogen, Cergy-Pontoise, France), 2-mercaptoethanol (2−10 M), (Invitrogen, Cergy-Pontoise, France), and 0.2% gentamicin (20 µg/ml) (Invitrogen, Cergy-Pontoise, France). The cells were stimulated with individual proteins or with peptide at a final concentration of 10 μg/ml for 10 days at 37°C in 5% CO_2_. Recombinant human IL-2 (BD Bioscience, San Diego, CA) (10 IU/well) was added at days 2, 4 and 7 during culture. For positive controls, cells were stimulated with phytohemagglutinin (PHA) (Sigma-Aldrich, St Louis, MO) (10 μg/ml) for 3 days. Negative control cells were not stimulated. Culture supernatants were then harvested and frozen at -80°C until use for ELISA, whereas cells were used for the ELISpot test.

### 2.16 Enzyme-linked immunospot assay

The ELISpot assay was performed with PBMCs stimulated for 10 days with proteins or peptides (10 μg/ml) or for 5 days with PHA (10 μg/ml) using a human IFN-γ immunospot assay. Briefly, 96-well PVDF filter plates (Millipore) were coated with 100 µl of anti-human IFN-γ monoclonal antibody (mAb) (2 µg/ml) (1-D1K, Mabtech) overnight at 4°C, and free binding sites were blocked with X-VIVO for 1 hour at room temperature. The cells were washed twice with phosphate-buffered saline (PBS 1X), resuspended in X-VIVO™ 15 medium (Lonza, Sweden), re-stimulated with protein or peptide (10 µg/ml) for 30 min at 37°C, and then transferred to coated ELISpot plates. The cells were incubated for 16 hours in a 5% CO2 humidified atmosphere at 37°C. Biotinylated anti-IFN-γ detection antibody (7-B6-1, Mabtech) (1 µg/ml) was added, and the plates were incubated for 2 hours at room temperature. After washing, ExtrAvidin-Alkaline Phosphatase (Sigma) diluted 1/2000 was added, and the plates were incubated for 30 min at room temperature. The spots were revealed by adding the substrate (BCIP/NBT)-containing buffer. Spots were counted using a CTL ImmunoSpot reader (CTL Analysers, Shaker Heights, OH). The results are expressed as spot-forming units (SFU) per 106 PBMCs.

### 2.17 Mouse immunization

Female BALB/c mice aged 6 to 8 weeks were used for the immunogenicity study. Each group of mice consisted of five animals. For peptides (P3/FUR/x3, P4/TMP/x3), animals were immunized via the subcutaneous or intramuscular route and received three injections at day 0, day 14, and day 28 with 200 μg of P3/FUR/x3 or P4/TMP/x3 mixed with 25 μg of CpG (ODN 2395 VacciGrade, InvivoGen, USA) adjuvant. PBS was used for the immunization of the control group. Blood samples were collected before immunization, then at days 28 and 42 and up to day 100 for some groups. For the recombinant protein subunits (P3-L, SJ/FT), we evaluated the effect of escalating doses (10, 25, and 50 μg), as well as the effect of adjuvants (2 μg of CpG, 5 μg of MPLA, and 50 μg of alhydrogel). Mice received two successive shots at day 0 and day 14, and blood samples were collected 14 days after the second administration. Serum samples were analyzed for specific IgG against antigens with an in-house indirect ELISA. Briefly, mouse sera were diluted at 1:100 and added to the coated plate with the designed peptide/protein antigens. After incubation, HRP-conjugated goat anti-mouse IgG diluted 1:2000 (Abcam) was used as the secondary antibody.

### 2.18 Plaque reduction neutralization test

Live virus experiments were performed in approved biosafety level 3 (BSL-3) facilities at Georgia State University and strictly followed the approved standard operation procedures. Neutralizing Abs were measured by plaque reduction neutralization assay (PRNT) using ancestral B.1 Wuhan virus (BEI# NR-52281). For PRNT assay Vero E6-TMPRSS2-T2A-ACE2 cells (BEI NR-54970) were seeded in 6-well plates at 200,000 cells/well in M199 medium with Earle’s salts (10X) supplemented with 5% inactivated fetal bovine serum, buffered with 3% sodium bicarbonate and 1% Penicillin-Streptomycin for 3 days (preformed monolayers). Serum samples were heated at 56oC for 1 hour to inactivate complement. Heat-inactivated serum samples were diluted at 1:10 and were further serially diluted 4-fold from 1:10 to 1:5120. 60μl of serially diluted serum was mixed in 96-well plates with an equal volume of 100 PFU of SARS-CoV-2. Serum/virus mixtures were incubated for 30 mins at 37°C. After incubation, 100 ul of serum/virus mixture was transferred to monolayered cells and incubated for 1 h at 37°C with gentle rocking every 15 minutes. After incubation, 2 ml of 1% low melting agarose media was added to each well and plates were incubated at 37°C for 2 days. After two days, plates were overlaid with 2% neutral red in 1% low-melting agarose for visualizing plaque formation. The number of plaques were counted and recorded in each well. The neutralization titer for each dilution was calculated as follows: NT = [1-(plaque count with sera/plaque count without sera)]x100.

### 2.19 Data analysis

For the analysis of the ELISA results from the cohort of COVID-19 patients, we used R (v. 4.0.5), readxl (v. 1.3.1), dplyr (v. 1.0.6) and stringi (v. 1.5.3) software packages for data handling and analysis. The “disease status” variable was assigned a numeral (integer) and factor (categorical variable) using the following rules: 0=asymptomatic, 1 = mild, 2 = moderate, and 3 = severe. We transformed the OD values obtained from the ELISA experiments from numeric (float, quantitative variable) to factor (categorical variable) data using the following rule: OD < 0.45 = immune (–) and OD ≥ 0.45 = immune (+) for the epitopes displayed by peptide P3/Fur/x3. For calculation of the chi-square test, we used the chisq.test function from the R Stats Package. We used the Monte Carlo simulation method instead of the asymptotic chi-squared distribution of the test statistic to compute the p value because some of the expected values were below 5. For the analysis of IFN-γ data, statistical analyses were performed with GraphPad Prism 9.2.0 (GraphPad Software, San Diego, CA). The results are expressed as medians (interquartile range, IQR, as variances). The Wilcoxon signed-rank test was used to compare the median IFN-γ production between stimulated and unstimulated cells. The Mann-Whitney test was used for intergroup analysis. p values less than 0.05 were considered to indicate significance. Correlations were estimated using the Spearman rank (rs) correlation coefficient.

## 3 Results

### 3.1 Identification of potential B- and T-cell epitopes in the SARS-CoV-2 S protein structural region involved in infectivity

**Figure 1:**
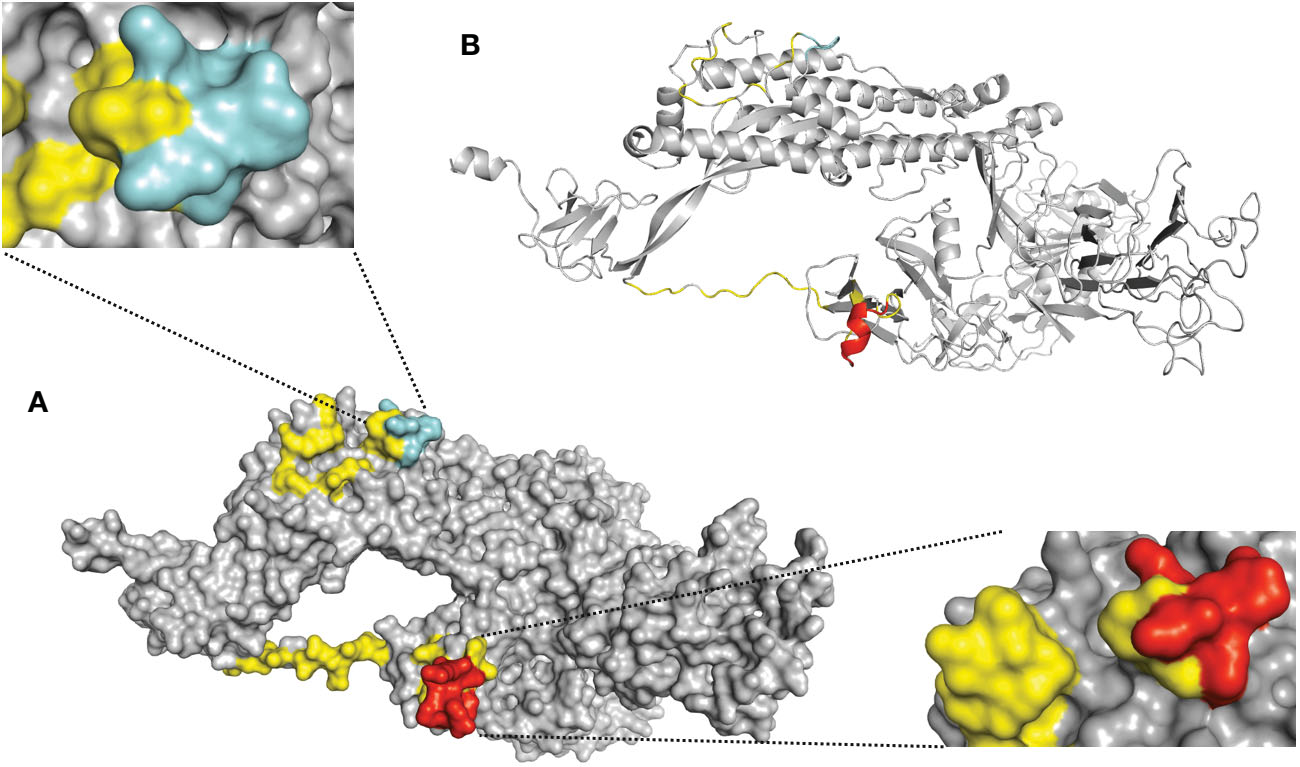
Solvent-exposed immunogenic regions of the SARS-CoV-2 S protein 3D model. Schematic representation of the S protein monomer extracted from the crystal structure of the trimeric form (PDB ID: 6VXX). **(A)** This panel is a surface representation of the S protein monomer. The solvent-exposed residues at the S1/S2 junction (residues E648-L809) are shown in yellow. The predicted antigenic peptides derived from the furin cleavage site loop are shown in red [lower right corner= side view], and the TMPRSS2 cleavage site loop is shown in cyan blue [upper left corner= top view]. **(B)** This panel is a cartoon representation of the S protein monomer. The loops corresponding to furin and TMPRSS2 are shown in red and cyan blue respectively.

**Figure 2:**
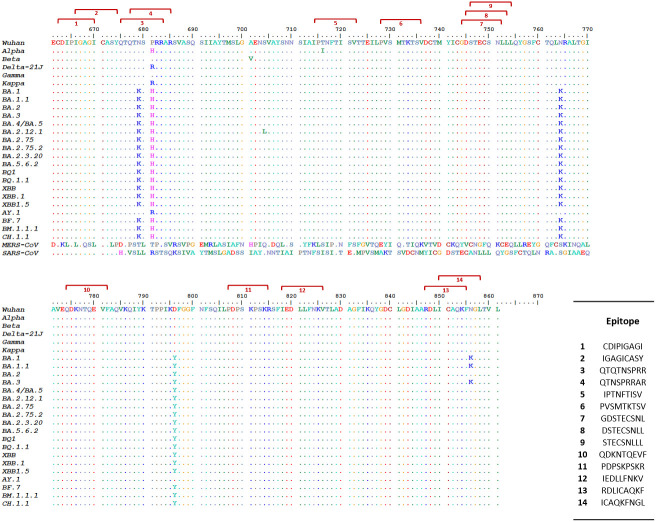
Predicted SARS-CoV-2 S protein S1/S2 junction immunogenic peptides and their cross-variant sequence conservation. This figure shows the alignment of *in silico* predicted immunogenic peptides [red brackets] generated by the proteasome/immunoproteasome of antigen-presenting cells (APCs) upon processing of the SARS-CoV-2 S protein S1/S2 junction sequence with the corresponding sequence of 24 SARS-CoV-2 variants. Peptides 3 and 4 contained within the solvent-exposed sequence Q662 to S676 (**Supplementary Table 1**) overlap with the PRRAR motif. Peptide 1 lies in the solvent-exposed sequence spanning residues E648 to G654. Peptide 11 overlaps with the TMPRSS2 cleavage site SKPSKR. We retrieved the amino acid sequence of the extracellular domains of SARS-CoV, MERS-CoV, and SARS-CoV-2 spike proteins from the National Center for Biological Information (NCBI) protein ID: YP_009825051.1, YP_009047204.1 and YP_009724390.1, respectively. We generated the SARS-CoV-2 variant sequences by introducing specific mutations into the Wuhan sequence according to published mutations in the CoVariants database (https://covariants.org/) and the Stanford University SARS-CoV-2 Variants database (https://covdb.stanford.edu/variants/omicron_ba_1_3/)].

The first step of our approach to designing a SARS-CoV-2 precision vaccine consisted of delimiting solvent-exposed sequences in the S protein S1/S2 subunit junction that potentially contain immunogenic epitopes. We extracted the structure of the SARS-CoV-2 spike protein monomer from the structural 3D model PDB ID: 6VXX using PyMOL software and visualized it using the Bepipred.2 server. We observed that the S1/S2 junction is mostly solvent exposed (**Figure 1**). Moreover, the *server* revealed potential B-cell epitopes in this structural region (**Supplementary Table 1**). This analysis also showed that furin and TMPRSS2 proteolytic motif sequences lie in two distant solvent-exposed loops (**Figure 1**) and are part of the predicted potential epitopes, as indicated by the estimated epitope probabilities (**Supplementary Table 1**). These observations suggest that this important region for virus infectivity can elicit an antibody response. We also submitted the SARS-CoV-2 S protein S1/S2 junction to the improved immunoproteasome cleavage-prediction server to search for putative T-cell epitopes. We identified fourteen immunogenic epitopes that the immunoproteasome of infected cells might generate upon processing of the SARS-CoV-2 S protein (**Figure 2**). To check whether these predicted epitope sequences are conserved across SAR-CoV-2 variants, we aligned them using BioEdit software (Hall, 1999). Thus, we ran the aligned sequences on the Epitope Conservancy Analysis tool of the freely accessible Immune-Epitope Database (IEDB). The percentage of protein sequence matches showed that these peptides are conserved among the twenty-four SARS-CoV-2 variants that circulated and/or are still circulating (**Supplementary Table 2**). These epitopes are not conserved in SARS-CoV or MERS-CoV (**Figure 2**). Peptides 3 and 4 contain residues of the FCS sequence (PRRAR), peptide 11 contains the TMPRSS2 cleavage motif, and the sequence of peptide 12 overlaps with the fusion peptide (FP) sequence (**Supplementary Table 2**).

For confirmation of the T-cell nature of these epitopes, we used the IFN epitope prediction server (Dhanda et al., 2013) to determine which epitopes potentially induce interferon γ (IFN−γ) . The results of this analysis identified peptides 3 and 11 as IFN-γ inducing epitopes (**Supplementary Table 3**).

To further investigate the immunogenic epitopes at SARS-CoV-2 for presentation to the immune system by MHC molecules, we used the Net MHC pan, ProPred I, NetMHC II and ProPred II servers to run algorithms that perform epitope prediction with evaluation of antigenic peptide binding to defined MHC I and II molecules. This study showed that numerous predicted peptides from the SARS-CoV-2 S protein S1/S2 junction bind MHC class 1 and class 2 molecules of known sequence (**Supplementary Tables 4–6**). Several of the predicted peptides contain or overlap amino acids from the FCS or the TMPRSS2 proteolytic site or lie near these sites. Some of these peptides were predicted to have strong binding affinity for specific MHC alleles, while others were predicted to have weak binding affinity (**Supplementary Tables 4–6**). This finding implies that the predicted epitopes can be presented to the immune system through numerous common MHC 1 and class 2 molecules.

The prediction data show that the SARS-CoV-2 S protein S1/S2 junction contains epitopes that can potentially elicit both T- and B-cell immune responses, including epitopes that overlap with the furin and TMPRSS2 proteolytic sites.

### 3.2 Study of the antibody response to predicted epitopes from S protein S1/S2 junction and to the RBD region in patients with COVID-19

To check if the predicted epitopes overlapping with the sequence involved in SARS-CoV-2 infectivity generate an immune response in patients with COVID-19, we used two synthetic peptides, P3/FUR/x3 and P4/TMP/x3. We designed these peptides by triplicating a sixteen-residue core sequence (Q662-I679) that spans the FCS in P3/FUR/x3 and a nine-residue core sequence (P794-R802) that spans the TMPRSS2 motif in P4/TMP/x3. In each polypeptide, we introduced the five-residue-long flexible sequence GGGGS as a molecular linker between each repeated core sequence (**Table 2**). This linker brings stability and maintains distance between epitopes for stable and correct folding of the synthetized polypeptide (Patel et al., 2022). We used these polypeptides to investigate the specific antibody response in patients with COVID-19.

**Table 2:** Design of antigenic polypeptides.

Peptide Name	Peptide Sequence
P3/FUR/x3	GGGGSQTNSPRRARSVASQSIGGGGSQTNSPRRARSVASQSIGGGGSQTNSPRRARSVASQSI
P4/TMP/x3	GGGGSPDPSKPSKRGGGGSPDPSKPSKRGGGGSPDPSKPSKR

Amino acid sequence of polypeptides (P3/FUR/x3) and (P4/TMP/x3). The triplicate core sequence for each polypeptide is shown in red. The GGGGS sequence is introduced at the NH2 terminus of each core sequence. These polypeptides can be structurally modified to introduce subtle conformational changes, including modifications of MHC-anchor substitutions, which increase their stability, protease resistance and immunogenicity and enhance their vaccinating potential. P3, Peptide 3; P4, Peptide 4.

**Figure 3:**
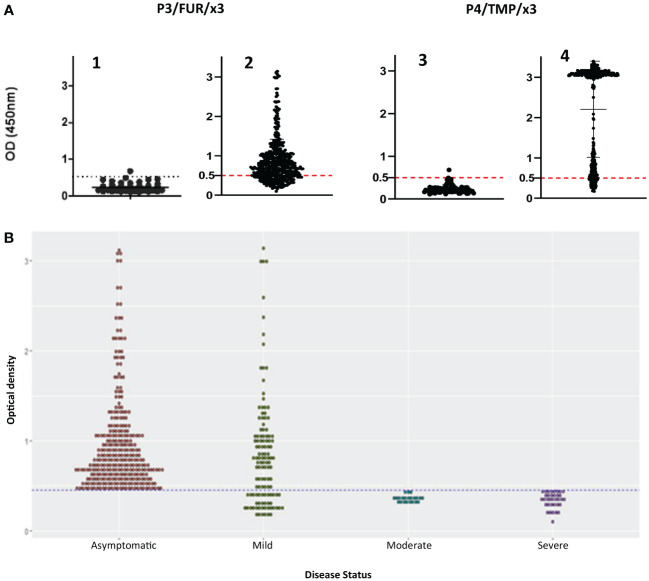
IgG response of patients with COVID-19 to synthetic peptides derived from S protein S1/S2 junction. **(A)** Results of ELISA using polypeptides P3/FUR/x3 and P4/TMP/x3 with sera of 500 COVID-19 patients(A2 and A4) and with healthy controls (A1 and A3). The cut-off (OD of 0.5) with a 95% CI (2 SDs) was calculated by testing 100 prepandemic negative control sera from healthy people. **(B)** Analysis of the data from the ELISA experiment using P3/FUR/x3 according to the patient’s disease status. The dot plot chart was produced using R and the ggplot2 (v. 3.3.3) library showing the distribution of “P3/FUR/x3 “OD at 450 nm” versus “disease status” (binwidth = 0.05).

To study this antibody response, we developed an indirect enzyme-linked immunosorbent assay (ELISA) using the P3/FUR/x3 and P4/TMP/x3 polypeptides as antigens and examined the cohort of 500 patients with COVID as described in **Table 1**, as well as 100 negative control serum specimens collected before the pandemic. The data showed that none of the control sera reacted with P3/FUR/x3 (**Figure 3**-A1) and P4/TMP/x3 (**Figure 3**-A3), while 80% and 86% of the COVID-19 patients’ sera specifically recognized P3/FUR/x3 (**Figure 3**-A2) and P4/TMP/x3 (**Figure 3**-A4), respectively. We detected this specific antibody response in numerous patients’ sera up to 50 weeks after infection. These data show that the S protein S1/S2 junction involved in SARS-CoV-2 infectivity elicits a natural robust and persistent antibody response.

To investigate the nature of this antibody response, we analyzed the ELISA data in the context of disease clinical forms. We observed a highly significant difference (p value = 0.0001) between patients’ antibody response to P3/FUR/x3 based on their disease status. All patients with the asymptomatic or mild form of the disease developed antibodies to epitopes in or around the furin cleavage site sequence (**Figure 3B**). Therefore, the antibody response to the S protein epitopes covering the furin proteolytic cleavage site in patients with COVID-19 significantly correlates with protective immunity to SARS-CoV-2.

Furthermore, the antibody response we observed did not cause antibody-dependent enhancement (ADE) of infection. Indeed, the patients who developed this type of immunity experienced rapid and full recovery, and no COVID-19 was recorded among them ten months after this study.

The study of the cohort of patients with COVI-19 for antibody response to the SARS-CoV-2 S protein RBD using a commercial ELISA kit revealed that 36.4% of the total samples had an IgG ELISA titer less than the cut-off (15 Unit/ml), indicating that these patients were negative for IgG against the RBD region at the time of blood sampling. Indeed, in this group of patients the blood sampling was performed at least 6 months after infection. Meanwhile, out of the 63.6% positive sera, 51.6% developed a low IgG titer, i.e less than three times the cut-off (45 Unit/ml); 18.3% a medium titer between 45 and 100 Unit/ml and 30.1% produced a high COVID-19 IgG titer above 100 Unit/ml. The highest IgG titer was 253.16 units/ml. No correlation was observed between the antibody response to the RDB and the response to the three candidate vaccinating antigens. There was also no correlation between the anti RDB IgG titer and the disease status.

### 3.3 Engineering and production of recombinant multiepitope vaccinating protein subunit candidates

**Figure 4:**
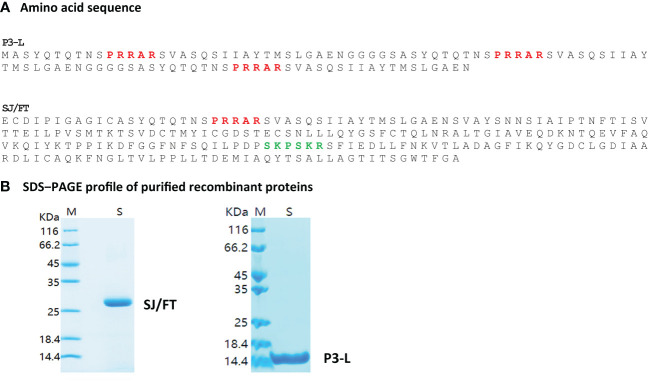
Engineered P3-L and SJ/FT vaccinating protein subunits. P3-L for Long, reference to the core antigenic sequence which is long longer (32 AA) than is P3 (16AA). SJ/FT stands for S protein, J, Junction; F, Furin; T, TMPRSS2. Red, Furin cut site; Green, TMPRSS2 cut site. **(A)** Amino acid sequence **(B)** SDS-PAGE profile of purified recombinant proteins.

Based on the data from the patient-centered study that showed strong and persistent natural immunity against the epitopes of the S protein covering the furin and TMPRSS2 cleavage sites, we developed two protein subunits, P3-L and SJ/FT, that encompassed the epitopes identified from the S protein S1/S2 junction. P3-L is 107 amino acids long, 11.1 kDa protein engineered by sequentially adding 3 core sequences of 32 amino acids that comprise the furin proteolytic site separated by a GGGGS molecular linker (**Figure 4A**). To design the SJ/FT protein subunit, we selected a sequence of 230 amino acids (24.8 kDa) from the S1/S2 junction that comprises both the furin and TMPRSS2 proteolytic motifs and includes the epitopes predicted earlier by computational analysis.

We selected these two subunits based on their predicted good antigenicity, no allergenicity and stability. Indeed, we used the VaxiJen server to predict the immunogenicity of S1/S2 junction-derived antigens P3/FUR/x3, P4/TMP/x3, P3-L and SJ/FT. The predicted immunogenicity data suggest that these polypeptides and protein subunits are immunogenic (**Supplementary Table 6**). In addition, the prediction of allergenicity using the AlgPred and AllerTOP computational tools showed that P3/FUR/x3, P4/TMP/x3 and subunits P3-L and SJ/FT are nonallergenic (**Table 3**), which makes them suitable for vaccination. Furthermore, we determined the physicochemical properties of P3-L and SJ/FT using the ProtParam tool of the EXPASY database server. The relatively low predicted instability index, good solubility values, and estimated half-life (**Supplementary Table 7**) suggest that the physicochemical properties of the engineered antigens are suitable for production as recombinant vaccine subunits. We produced P3-L and SJ/FT as recombinant proteins in *E. coli* (**Figure 4B**) and purified them as endotoxin-free proteins for use in immunization studies.

**Table 3:** Predicted antigenicity and allergenicity of SARS-CoV-2 polypeptide and protein subunit vaccines.

A-Immunogenicity Prediction				
Subunit	P3/FUR/x3	P4/TMP/x3	P3-L	SJ/FT
VAXIJEN	0.4331	0.6850	0.4436	0.4512
B-Allergenicity Prediction				
Subunit	P3/FUR/x3	P4/TMP/x3	P3-L	SJ/FT
AllerTOP	(-)	(+)	(+)	(-)
AlgPred	(-) ^#^*0.5130	(-)*	(-) ^#^ *0.7027	(-) ^®^*0.5310

#Prediction by SVM method based on amino acid composition [score threshold=-0.4].

^®^Prediction based on SVM method based on dipeptide composition [score threshold= -0.2].

*The protein sequence does not contain an experimentally proven IgE epitope.

### 3.4 Antibody and cellular immune response to engineered vaccinating antigens candidates of patients with COVID-19

**Figure 5:**
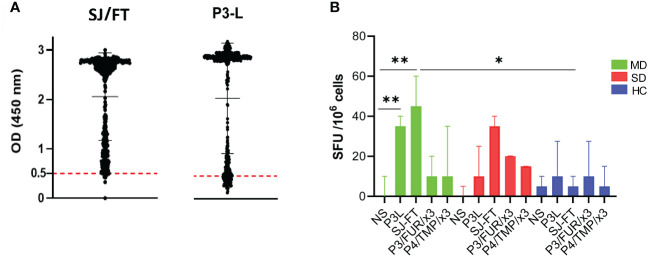
IgG and T-cell response of patients with COVID-19 to SARS-CoV-2 S protein junction-derived antigens: **(A)** Results of the ELISA experiment with protein subunits SJ/FT and P3-L and the sera from 500 COVID-19 patients. **(B)** IFN-γ production analysis: PBMCs from COVID-19 patients with mild disease (MD, green), COVID-19 patients with severe disease (SD, red) or healthy controls (HC, blue) were stimulated with S protein junction-derived proteins P3-L or SJ/FT and polypeptides P3/FUR/x3 or P4/TMP/x3 at a final concentration of 10 µg/ml. At 10 days, cells were collected and ELISpot was used to measure IFN-γ production by. Histograms represent the median with interquartile range. Statistical analysis: * indicates a significant difference at *p*<0.05 and ** p<0.001.

To further investigate the vaccinating potential of the engineered recombinant subunits, we analyzed antibody and T cell responses in patients with COVID-19 antibodies to these antigens. ELISA experiments using P3-L and SJ/FT protein subunits as antigens and the serum of the patients of the COVID-19 cohort showed specific antibody responses in 84.7% and 97%, respectively (**Figure 5A**). To investigate the T-cell response, we studied IFN-γ production upon stimulation of the patients’ immune cells using P3-L and SJ/FT. We used PBMCs from COVID-19 patients with mild disease (MD) or severe disease (SD) and healthy controls (HCs) as defined by the WHO to perform ELISpot experiments. The results showed that the number of cells producing IFN-γ in response to stimulation with the P3-L and SJ/FT proteins, compared to unstimulated cells (NS), was highest for patients with MD (*p*<0.001) (**Figure 5B**). These observations show that the P3-L and SJ/FT protein subunits contain epitopes that generate antibody and T-cell responses in patients with COVID-19.

### 3.5 Modelling of the engineered candidate vaccinating antigen structures

**Figure 6:**
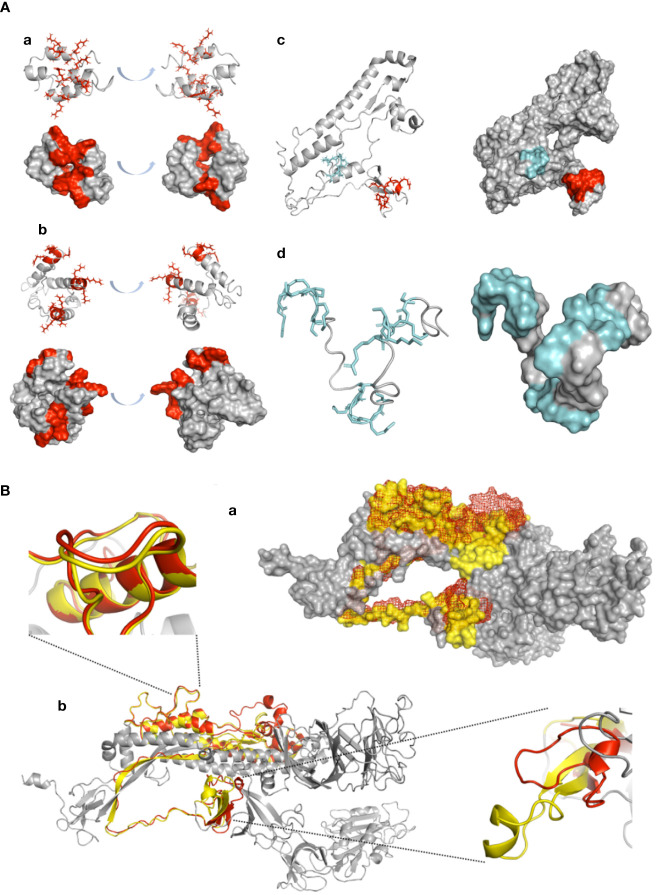
Confirmation of antigenic structure of designed vaccine candidates and conservation by ab initio structure prediction. **(A)** 3D modelling: Designed peptides were modelled using the ITASSER ab initio structure prediction server. Models are displayed in cartoon representation. Surface representation is shown for all peptides, with the coloured surface representing antigenic epitopes. PRRAR furin cleavage site residue side chains are displayed in red, and the TMP cleavage site is displayed in cyan. All models are represented in two side views with 180° angle rotation. (a) P3/Fur/x3, (b) P3-L, (c) SJ/FT and (d) P4/TMP/x3. **(B)** SJ/FT recombinant vaccine subunit *ab initio* model superimposition with the 3D SARS-CoV-2 S protein model. (a) SJ/FT model (red) superimposed with the full spike protein homology model (grey/yellow) generated by the Swiss-PDB server. SJ/FT structure is shown in mesh representation, spike structure shown in surface representation. (b) Cartoon representation. Residues within predicted antigenic loops are shown for the furin cleavage site (lower right corner/top view) and TMPRSS2 cleavage site (upper left corner/side view). The predicted SJ/FT model is shown in red, and the predicted spike protein model is shown in yellow. The total RMSD for 171 atoms is 0.917 Å.

To further assess the vaccinating potential of the P3-L and SJ/FT protein subunits and polypeptides P3/FUR/x3 and P4/TMP/x3, we carried out a computational study to check whether these antigens exhibit a conserved or mimicked native 3D structure of the SARS-CoV-2 S protein S1/S2 junction. We constructed 3D models of their structures and compared them with the native 3D structure of the S protein monomer. The predicted structural models show an alpha coil/helix fold that is consistent with the structure of immunogenic epitopes and solvent-accessible protein sequences. A repetitive 3-fold display of the alpha coil/helix fold is observed in the P3/FUR/x3 peptide and protein subunit P3-L (**Figure 6A**), which shows that the unit peptide sequence containing the PRRAR epitope folds independent of the GGGGS linker. Superimposition of the SJ/FT and S protein 3D models (**Figure 6B**) shows that the overall conformation of the sequence containing the immunogenic epitopes is very well conserved with an observed RMSD of 0.912 angstrom and 171 amino acids out of 230 superimposing perfectly on the native S protein corresponding sequence.

The data we gathered suggest strongly that the antigen-presenting cells process P3-L, SJ/FT and P3/FUR/x 3 engineered vaccine candidates’ antigens, the same way they do with the SARS-CoV-2 native S protein. The data also support their use as candidate vaccinating antigens.

### 3.6 BALB/c mouse IgG response to synthetic polypeptides P3/FUR/x3 and P4/TMP/x3 and protein subunits P3-L and SJ/FT

**Figure 7:**
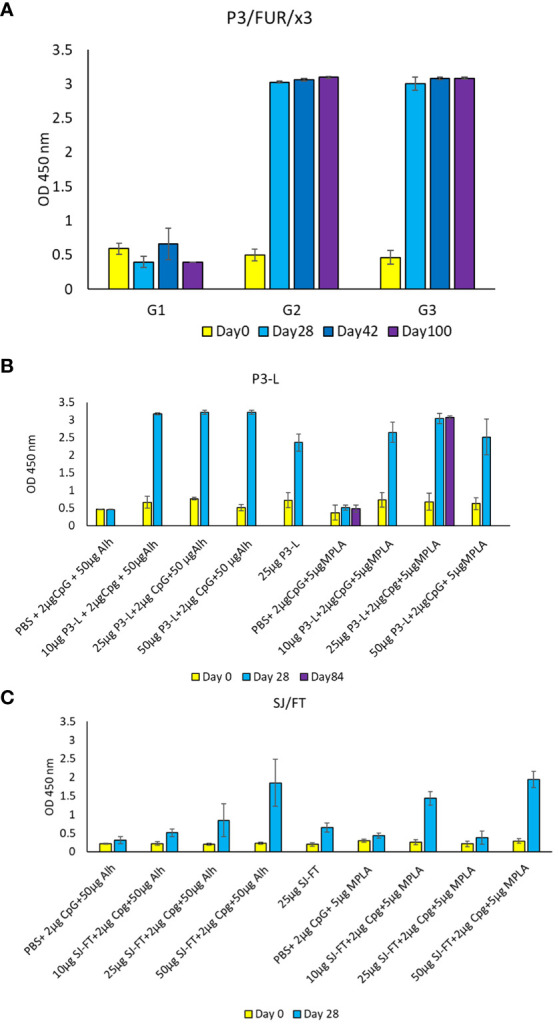
IgG response of BALB/c mice immunized using the engineered polypeptides and protein subunits. **(A)** Mouse antibody response [OD values] after immunization with the polypeptide P3/Fur/x3. BALB/c mice received 3 doses of 200 µg mixed with 25 µg of CpG at day 0, day 14 and day 28. Blood samples were collected at day 0, day 28, day 42 and day 100. Control mice received PBS. **(B, C)** Mouse antibody response [OD values] after immunization with the recombinant subunits P3-L and SJ/FT. BALB/c mice received 3 doses (10 µg, 25 µg or 50 µg) mixed with 2 µg of CpG and 50 µg of Alh or 2 µg of CpG and 5 µg of MPLA at day 0 and day 14. Blood samples were collected at day 0, day 28 and day 84. Control mice received PBS.

To assess the vaccinating potential of the engineered antigens *in vivo*, we immunized BALB/c mice with different concentrations of P3-L, SJ/FT, P3/FUR/x3 and P4/TMP/x3 ranging from 25 µg to 200 μg. We also used various adjuvants alone or in combination to determine the best adjuvant to use in vaccination. We immunized different groups of 5 mice each by administering 2 doses of the antigens within an interval of two weeks (**Supplementary Table 8**). We collected sera from week 4 to week 14 following immunization. The results of ELISA performed with the sera collected following immunization of mice with 200 µg of the polypeptide P3/FUR/x3 showed the highest antibody response in the group of mice administered the CpG TLR9 ligand adjuvant (**Figure 7A**). In mice immunized using the P3-L and SJ/FT protein subunits, we observed the highest IgG titres with a combination of CpG and either one of the adjuvants alhydrogel or MPLA (**Figures 7B, C**). P4/TMP/x3 did not induce repeatedly a significant IgG response [data not shown]

### 3.7 Neutralizing activity of the antibody response to the candidates vaccinating antigens

We evaluated the neutralization capacity of two pool of sera samples collected from COVID-19 patients with respectively low and medium/high antibody titers against vaccinating antigens but in which antibodies against RBD antigens were not detected. The pool of sera with low antibody titer was from patients with the moderate form of the disease while the pool of sera with the medium/high antibody titer was from asymptomatic patients.

**Figure 8:**
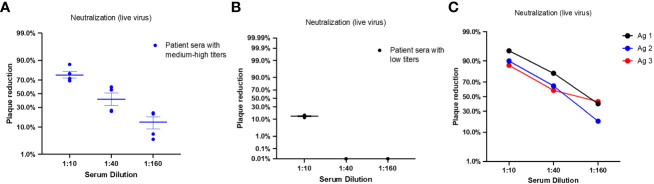
Neutralizing antibody titers of patients with COVID-19 and immunized BALB/c mice for candidates vaccinating antigens. Each dot represents one sample; middle horizontal bar represents the mean and error bars are SEM. **(A)** NAb titers were measured by PRNT assay against SARS-CoV-2 infectious virus in sera samples pooled from patients with negative RBD antibodies and medium to high antibody levels against vaccinating antigens. Serum dilutions reducing the plaque percentage are presented (n=4). **(B)** NAb titers were measured by PRNT assay against SARS-CoV-2 infectious virus in sera samples pooled from patients with negative RBD antibodies and low antibody titer against vaccinating antigens. Serum dilutions reducing the plaque percentage are presented (n=3). **(C)** NAb titers were measured by PRNT assay against SARS-CoV-2 infectious virus in sera samples pooled from mice vaccinated with Ag1/P3 (black), Ag2/P3-L (blue), or Ag3/SJ/FT (red). Serum dilutions showing the plaque percentage reduction are displayed in the x axis.

Neutralizing Ab measurements based on PRNT assay revealed that patient sera with medium to high titers against vaccinating antigens have the capacity to neutralize SARS-CoV-2 infectious virus (B.1) and demonstrated an average plaque reduction of 76.5% at 1:10 serum dilution (**Figure 8A**). Consistent with the low antibody levels against vaccinating antigens in some patient sera samples, we detected low neutralization activity against SARS-CoV-2 infectious with an average plaque reduction of 13.7% at 1:10 serum dilution (**Figure 8B**). The sera in this pool were from patient with the moderate form of the disease.

Next, we evaluated sera collected from vaccinated mice 28 days post immunization for neutralization capacity. Neutralizing Ab measurements based on PRNT assay revealed that the three vaccinating antigens elicited antibodies with efficacy to neutralize SARS-CoV-2 infectious virus. High NAb responses were detected at 1:10 dilution with an average plaque reduction of 90.7%. Sera from mice vaccinated with Ag1 (P3) showed the highest neutralization capacity with 95% plaque reduction at 1:10 dilution. Sera from mice vaccinated with Ag2 (P3-L) and Ag3 (SJ/FT) showed 90% and 87% plaque reduction, respectively. Notably, moderate plaque reduction was still detected at 1:160 serum dilution for all three samples (**Figure 8C**).

Through the development of this new approach for designing precision viral vaccines, we used computational tools to generate a data set that identified SARS-CoV-2 immunogenic epitopes in the virus structural region associated with infectivity. We experimentally linked these epitopes to protective immunity in patients with COVID-19. Based on these findings, we engineered vaccinating subunit antigens that we thoroughly tested *in vivo* by immunizing mice. Through this vaccination study, we determined the best antigen concentration and adjuvants that elicited a robust neutralizing antibody response.

## 4 Discussion

In this paper, we describe the different steps of a new approach to design precision vaccines through its application to SARS-CoV-2. Knowing that most of the highly pathogenic human zoonotic viruses that can cause pandemics have structural features associated with infectivity (Izaguirre, 2019), we designed this approach to engineer vaccinating antigens that mimic the viral surface structure involved in infectivity. For SARS-CoV-2, we engineered polypeptides and recombinant protein subunits that mimic predicted immunogenic sequences present at the S protein S1/S2 junction. This region contains the sites of furin and TMPRSS2 proteolytic cleavage that activate the virus and enhance infectivity (Fuentes-Prior, 2021). We found that the engineered antigens were targeted by the natural immune response in patients with COVID-19 who developed protective immunity. The patient’s antibody response to the engineered candidate vaccinating antigens was most likely triggered by the live virus thus the native trimeric form of the S protein. This is highly suggested by asymptomatic patients that were anti RBD antibody negative but still positive for P3, P4, P3-L and SJ/FT engineered antigens. Mouse immunization assays showed that three out the four designed antigens elicited a strong neutralizing antibody response. These observations are in favor of their vaccinating potential. Our findings are supported by Li et al. report of the presence in a Chinese patient with CoVID-19 of a specific anti furin cleavage site antibody that protected mice from SARS-CoV-2 infection (Li et al., 2023). In addition, Schwarz and collaborators (Schwarz et al., 2021) reported through a proteome wide analysis an antibody response signature in patients with the mild form of COVID-19 that involves two peptides containing the Furin and TMPRSS2 cleavage motifs.

The prediction of immunogenic epitopes in the viral surface protein associated with infectivity is an important step of this approach to designing precision vaccines. Moreover, the use of a patient-centered study is crucial to determine whether the predicted epitopes generate natural IgG and T-cell natural immunity. As observed in this work, the ELISA patient-centered study using antigens containing the predicted epitopes, was instrumental in uncovering a neutralizing antibodies response that correlate the disease status, which is in favor of the vaccinating potential of these epitopes in humans. Indeed, while the neutralization of antibodies directed to the RBD epitopes is based on interfering with binding to the ACE2 receptor according to the classification of the RBD epitopes and the corresponding antibodies classes (Deshpande et al., 2021; Felbinger N et al., 2023), the approach we developed targets non-glycosylated epitopes outside of the RBD that are not involved in the binding to ACE2. Thus, the neutralization is in principle based upon preventing the proteolytic activity responsible of the virus activation which mitigate infectivity. Such approach can be considered for other viruses whose pathogenicity is contingent to proteolytic activation. Focusing the search for immunogenic epitopes on the viral structure associated with infectivity is the hallmark of this precision vaccine rational design. It is the precise immune targeting that distinguishes this new approach from conventional approaches for vaccine development. Indeed, all the first COVID-19 vaccines use a full-length S protein as a vaccinating antigen and generate non-precision immunity mainly directed to the immunodominant although genetically variable RBD epitopes (Bestle et al., 2020; Piccoli et al., 2020; Chen et al., 2021), which explains their decreased efficiency with SARS-CoV-2 genetic variants. Furthermore, the S protein is a biologically active protein that binds the ACE2 receptor and can interfere with associated physiological processes, particularly the renin/angiotensin system (RAS) (Wiese et al., 2020), causing unwanted side effects such as platelet aggregation, thrombosis and exacerbated inflammation (Angeli et al., 2021). In addition, immunization using a full-length protein-based approach is more likely to lead to ADE, as reported in SARS-C0V-2 (Karthik et al., 2020; Lee et al., 2020) and MERS-CoV (Prompetchara et al., 2020) infections.

Other vaccines in development are also using the entire S protein in the prefusion state (Pallesen et al., 2017; Ciaramella and Himansu, 2020) or the RBD for immunization. Such vaccines are no precision vaccines, and even though they are expected to be at least as efficient as the current vaccines, they will probably share their limitations. By targeting selected epitopes, the approach we developed in this study achieves precision and overcomes the genetic variation issue. Furthermore, our findings show that protection from the disease correlates with the natural immunity that targets the viral structure involved in infectivity. This highlights the importance of careful selection of vaccinating antigens for the development of efficient precision vaccines. This is in line with Zinkernagel’s theory stating that protective immunity is antigen-driven and not due to so-called “memory” B and T cells. Protective immunity is rather driven by periodical re-exposure to the right antigen (Zinkernagel and Hengartner, 2006). This makes vaccination using a precision vaccine the best measure for achieving large community protection beside natural herd immunity.

The engineering of vaccinating antigens that include the identified epitopes is a pivotal step of this approach for designing precision vaccines. We designed the vaccinating antigens as recombinant proteins that mimic the original viral antigenic structure by keeping the protein native scaffold. In addition, their biological and physicochemical properties, particularly allergenicity (Saha and Raghava, 2006) and molecular stability, were suitable for use in vaccine preparations. We also used homopolymerization for the design of the selected epitopes. In this engineering approach the sequences of an epitope are repeated several times within the antigen amino acid sequence. This design enhances immunogenicity (Borras-Cuesta et al., 1988). At this step of this approach to designing precision viral vaccines, a large array of engineering techniques is available (Zhou et al., 2020; Iyer et al., 2022). Multiple advanced delivery platforms can also be used to develop vaccinating antigens, particularly recombinant viral shuttle vectors or mRNA-mediated vaccination (Kremer, 2020; Polack et al., 2020), depending on the ease and cost of production, storage conditions and safety profile.

Animal immunization of the engineered antigens and testing of adjuvants are carried out in the final and mandatory step of the approach. In our study, the observed neutralizing robust IgG response, demonstrates that the selected epitopes are processed properly by the animal immune system and that the antigens we designed are good vaccinating candidates. For SARS-CoV-2, few animal models, such as ferrets, are available (Gimenes Lima et al., 2022). The ultimate testing of the vaccinating potential of engineered antigens is a challenge using live virus. This necessitates access to an adequate high-confinement animal facility, which may limit the simultaneous testing of various approaches of antiviral precision vaccines. Another limitation of this approach is the necessity to know the precise molecular basis of infectivity and pathogenicity before designing a precision vaccine for a given virus. This condition was fulfilled for the SARS-CoV-2 virus.

While precision vaccines are the best alternative to currently available COVID-19 vaccines, the new approach described in this report offers a way to develop efficient and precise antiviral candidate antigens vaccines for a number of highly pathogenic viruses in a shorter timeframe and at reasonable cost.

## Data availability statement

The original contributions presented in the study are included in the article/supplementary material. Further inquiries can be directed to the corresponding author.

## Ethics statement

The studies involving humans were approved by The Arabian Gulf University (Manama, Bahrain) approved the protocol (reference E031-PI-12/20). The studies were conducted in accordance with the local legislation and institutional requirements. The participants provided their written informed consent to participate in this study. The animal study was approved by The Arabian Gulf university (Manama, Bahrain) Research and Ethics committee. The study was conducted in accordance with the local legislation and institutional requirements.

## Author contributions

KT: Data curation, Investigation, Methodology, Supervision, Validation, Visualization, Writing – original draft. NB: Data curation, Methodology, Software, Visualization, Writing – original draft. AR: Data curation, Investigation, Methodology, Validation, Writing – original draft. AE: Data curation, Investigation, Methodology, Writing – original draft. DA: Data curation, Software, Validation, Visualization, Writing – original draft. SC: Data curation, Formal Analysis, Software, Validation, Writing – original draft. TB: Data curation, Investigation, Validation, Writing – original draft. MB: Investigation, Methodology, Writing – original draft. MK: Methodology, Investigation, Supervision, Writing – original draft, Writing – review & editing. M-DF: Conceptualization, Data curation, Formal Analysis, Funding acquisition, Methodology, Project administration, Resources, Software, Supervision, Validation, Writing – original draft, Writing – review & editing.
