# Supplementary material for: A novel approach to designing viral precision vaccines applied to SARS-CoV-2
Source: Front Cell Infect Microbiol. 2024 Apr 2;14:1346349. doi: 10.3389/fcimb.2024.1346349 (PMC11018900; doi:10.3389/fcimb.2024.1346349)
Supplement: Supplementary file 1 [file Table_1.docx]

**SUPPLEMENTARY TABLE 1: Prediction of the solvent accessibility of the SARS-CoV-2 S protein S1/S2 subunit junction by BepiPred-2.0 (Sequential B-Cell Epitope Predictor)**.

Green=solvent-exposed sequences predicted to contain B-cell epitopes. Red=epitopes containing the furin (PRRAR) and/or TMPRSS2 (SKPSK) cleavage sites.

AA=amino acid, E=exposed, B=buried, RSA=relative surface accessibility, Prob. =probability

| **Position** | **AA** | **E/B** | **RSA** | **Helix Prob.** | **Sheet Prob.** | **Coil Prob.** | **Epitope Prob.** |
| --- | --- | --- | --- | --- | --- | --- | --- |
| 648 | E | E | 0.55 | 0.022 | 0.552 | 0.426 | **0.56256** |
| 649 | C | B | 0.072 | 0.022 | 0.359 | 0.619 | **0.55878** |
| 650 | D | E | 0.608 | 0.021 | 0.279 | 0.699 | **0.53844** |
| 651 | I | E | 0.34 | 0.056 | 0.142 | 0.802 | **0.516** |
| 652 | P | E | 0.304 | 0.052 | 0.084 | 0.864 | **0.49978** |
| 653 | I | B | 0.226 | 0.056 | 0.142 | 0.802 | **0.47911** |
| 654 | G | E | 0.385 | 0.056 | 0.142 | 0.802 | **0.474** |
| 655 | A | B | 0.21 | 0.056 | 0.142 | 0.802 | 0.46133 |
| 656 | G | B | 0.085 | 0.069 | 0.386 | 0.545 | 0.44933 |
| 657 | I | B | 0.081 | 0.079 | 0.592 | 0.329 | 0.45411 |
| 658 | C | B | 0.028 | 0.074 | 0.484 | 0.442 | 0.46522 |
| 659 | A | B | 0.28 | 0.064 | 0.216 | 0.721 | 0.48833 |
| 660 | S | E | 0.406 | 0.052 | 0.084 | 0.864 | 0.52478 |
| 661 | Y | B | 0.311 | 0.053 | 0.043 | 0.903 | 0.55044 |
| 662 | Q | E | 0.365 | 0.018 | 0.088 | 0.893 | **0.57533** |
| 663 | T | E | 0.361 | 0.02 | 0.205 | 0.775 | **0.604** |
| 664 | Q | E | 0.449 | 0.022 | 0.359 | 0.619 | **0.61522** |
| 665 | T | E | 0.409 | 0.021 | 0.451 | 0.528 | **0.63322** |
| 666 | N | B | 0.266 | 0.022 | 0.552 | 0.426 | **0.634** |
| 667 | **S** | E | 0.372 | 0.022 | 0.552 | 0.426 | **0.62044** |
| 668 | **P** | B | 0.265 | 0.022 | 0.552 | 0.426 | **0.63311** |
| 669 | **R** | E | 0.345 | 0.021 | 0.451 | 0.528 | **0.62922** |
| 670 | **R** | E | 0.389 | 0.021 | 0.451 | 0.528 | **0.63478** |
| 671 | **A** | B | 0.235 | 0.022 | 0.359 | 0.619 | **0.63556** |
| 672 | **R** | E | 0.416 | 0.022 | 0.359 | 0.619 | **0.637** |
| 673 | S | E | 0.559 | 0.02 | 0.205 | 0.775 | **0.62756** |
| 674 | V | E | 0.537 | 0.019 | 0.141 | 0.84 | **0.60311** |
| 675 | A | E | 0.364 | 0.02 | 0.205 | 0.775 | **0.57** |
| 676 | S | E | 0.411 | 0.021 | 0.279 | 0.699 | **0.55611** |
| 677 | Q | B | 0.199 | 0.004 | 0.514 | 0.481 | 0.52 |
| 678 | S | B | 0.14 | 0.021 | 0.756 | 0.223 | 0.49722 |
| 679 | I | B | 0.118 | 0.018 | 0.846 | 0.136 | 0.48333 |
| 680 | I | B | 0.142 | 0.018 | 0.846 | 0.136 | 0.49389 |
| 681 | A | B | 0.113 | 0.021 | 0.756 | 0.223 | 0.49744 |
| 682 | Y | B | 0.167 | 0.021 | 0.451 | 0.528 | 0.52367 |
| 683 | T | E | 0.308 | 0.021 | 0.279 | 0.699 | **0.55233** |
| 684 | M | E | 0.318 | 0.019 | 0.141 | 0.84 | **0.58244** |
| 685 | S | E | 0.412 | 0.018 | 0.088 | 0.893 | **0.61144** |
| 686 | L | E | 0.386 | 0.052 | 0.084 | 0.864 | **0.64167** |
| 687 | G | E | 0.407 | 0.056 | 0.142 | 0.802 | **0.65711** |
| 688 | A | E | 0.398 | 0.056 | 0.142 | 0.802 | **0.67133** |
| 689 | E | E | 0.332 | 0.064 | 0.216 | 0.721 | **0.66878** |
| 690 | N | E | 0.378 | 0.021 | 0.279 | 0.699 | **0.67690** |
| 691 | S | E | 0.334 | 0.021 | 0.279 | 0.699 | **0.66289** |
| 692 | V | B | 0.238 | 0.022 | 0.359 | 0.619 | **0.64889** |
| 693 | A | E | 0.395 | 0.021 | 0.279 | 0.699 | **0.63422** |
| 694 | Y | E | 0.329 | 0.019 | 0.141 | 0.84 | **0.61322** |
| 695 | S | E | 0.382 | 0.02 | 0.205 | 0.775 | **0.55722** |
| 696 | N | E | 0.352 | 0.005 | 0.336 | 0.66 | **0.53678** |
| 697 | N | B | 0.195 | 0.004 | 0.514 | 0.481 | 0.48589 |
| 698 | S | B | 0.241 | 0.002 | 0.816 | 0.182 | 0.45978 |
| 699 | I | B | 0.044 | 0.001 | 0.9 | 0.099 | 0.44489 |
| 700 | A | B | 0.233 | 0.001 | 0.9 | 0.099 | 0.43922 |
| 701 | I | B | 0.078 | 0.023 | 0.655 | 0.322 | 0.43211 |
| 702 | P | B | 0.109 | 0.005 | 0.336 | 0.66 | 0.43644 |
| 703 | T | B | 0.283 | 0.019 | 0.141 | 0.84 | 0.43644 |
| 704 | N | E | 0.459 | 0.019 | 0.141 | 0.84 | 0.46111 |
| 705 | F | B | 0.149 | 0.022 | 0.359 | 0.619 | 0.46067 |
| 706 | T | E | 0.33 | 0.021 | 0.756 | 0.223 | 0.48211 |
| ------- | ------ | ------ | ------ | ------ | ------ | ------ | ------ |
| 778 | T | B | 0.161 | 0.056 | 0.142 | 0.802 | 0.585444 |
| 779 | P | E | 0.375 | 0.019 | 0.141 | 0.84 | **0.584778** |
| 780 | P | E | 0.436 | 0.02 | 0.205 | 0.775 | **0.587444** |
| 781 | I | B | 0.208 | 0.021 | 0.279 | 0.699 | **0.601778** |
| 782 | K | E | 0.459 | 0.02 | 0.205 | 0.775 | **0.597556** |
| 783 | D | E | 0.417 | 0.019 | 0.141 | 0.84 | **0.603778** |
| 784 | F | B | 0.21 | 0.018 | 0.088 | 0.893 | **0.604444** |
| 785 | G | E | 0.441 | 0.018 | 0.047 | 0.935 | **0.587667** |
| 786 | G | E | 0.456 | 0.018 | 0.088 | 0.893 | **0.584333** |
| 787 | F | B | 0.156 | 0.021 | 0.279 | 0.699 | **0.570889** |
| 788 | N | E | 0.312 | 0.022 | 0.359 | 0.619 | **0.556778** |
| 789 | F | B | 0.087 | 0.021 | 0.451 | 0.528 | 0.548333 |
| 790 | S | E | 0.333 | 0.022 | 0.359 | 0.619 | 0.544111 |
| 791 | Q | B | 0.261 | 0.022 | 0.359 | 0.619 | 0.547778 |
| 792 | I | B | 0.107 | 0.021 | 0.451 | 0.528 | 0.555889 |
| 793 | L | B | 0.169 | 0.022 | 0.359 | 0.619 | 0.564889 |
| 794 | P | E | 0.319 | 0.019 | 0.141 | 0.84 | 0.591556 |
| 795 | D | E | 0.486 | 0.018 | 0.047 | 0.935 | **0.610778** |
| 796 | P | E | 0.467 | 0.018 | 0.019 | 0.964 | **0.613579** |
| 797 | S | E | 0.484 | 0.018 | 0.047 | 0.935 | **0.621111** |
| 798 | K | E | 0.441 | 0.018 | 0.047 | 0.935 | **0.614556** |
| 799 | P | E | 0.406 | 0.053 | 0.043 | 0.903 | **0.598556** |
| 800 | S | E | 0.525 | 0.053 | 0.043 | 0.903 | **0.570111** |
| 801 | K | E | 0.293 | 0.191 | 0.086 | 0.723 | **0.533556** |
| 802 | R | B | 0.177 | 0.307 | 0.165 | 0.527 | 0.498556 |
| 803 | S | B | 0.138 | 0.321 | 0.252 | 0.427 | 0.472444 |
| 804 | F | B | 0.092 | 0.453 | 0.248 | 0.299 | 0.448333 |
| 805 | I | B | 0.052 | 0.578 | 0.229 | 0.194 | 0.425111 |
| 806 | E | B | 0.174 | 0.538 | 0.173 | 0.289 | 0.417111 |
| 807 | D | B | 0.271 | 0.502 | 0.102 | 0.396 | 0.424889 |
| 808 | L | B | 0.127 | 0.502 | 0.102 | 0.396 | 0.424333 |
| 809 | L | B | 0.11 | 0.386 | 0.097 | 0.517 | 0.422778 |

**SUPPLEMENTARY TABLE 2: Analysis of the conservation of the SARS-CoV-2 S protein S1/S2 junction 14 predicted proteasome/immunoproteasome epitopes in twenty-four viral genetic variants.**

**
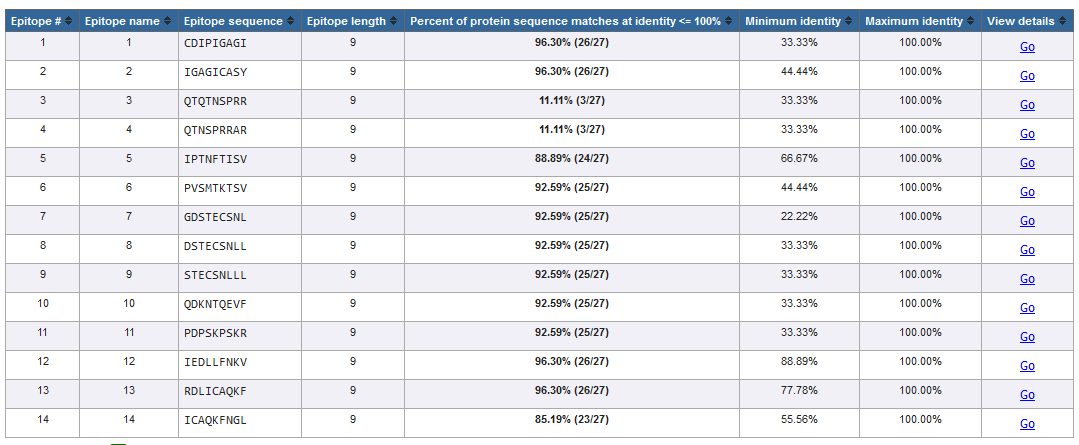
**

**SUPPLEMENTARY TABLE 3: Prediction of the IFN-γ-inducing epitopes among fourteen selected immunogenic epitopes from the SARS-CoV-2 S protein S1/S2 junction sequence.**

**
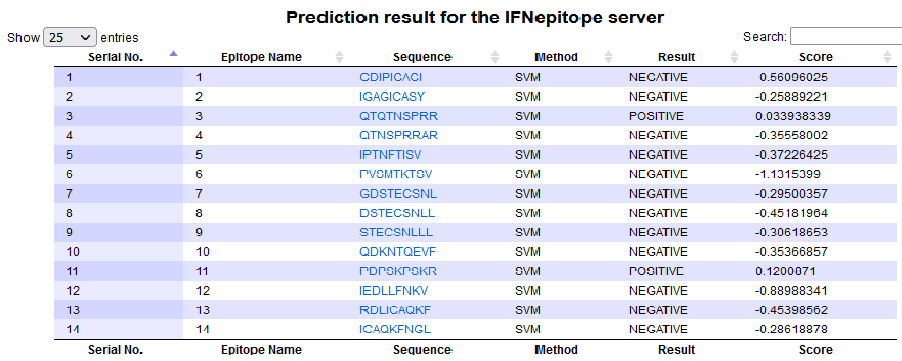
**

**SUPPLEMENTARY TABLE 4:**  **Prediction of antigenic peptides spanning the SARS-CoV-2 S protein S1/S2 junction binding to MHC class 1 peptide binding by the NetMHC pan server.**

**MHC =** MHC allele name.

**BindLevel=** (SB: strong binder, WB: weak binder). The peptide was identified as am SB if the % Rank was below the specified threshold (by default, 0.5%). The peptide was identified as a WB if the % Rank was above the threshold of the SBs but below the specified threshold for the WBs (by default, 2%). Red= Peptides that overlap or comprise amino acids of the furin or TMPRSS2 cleavage sites. Peptides that bind MHC alleles with high affinity (SB) are highlighted in yellow.

| **MHC** | **Peptide** | **Binding Level** |
| --- | --- | --- |
| HLA-A*03:01 | ASYQTQTNSPR | WB |
| HLA-A*03:01 | QTNSPRRAR | WB |
| HLA-B*07:02 | QTNSPRRARSVA | WB |
| HLA-B*07:02 | TNSPRRARSVA | WB |
| HLA-B*08:01 | NSPRRARSV | WB |
| HLA-B*07:02 | NSPRRARSVA | WB |
| HLA-B*08:01 | SPRRARSVA | WB |
| HLA-B*07:02 | SPRRARSVAS | WB |
| HLA-B*07:02 | SPRRARSVASQ | WB |
| HLA-B*07:02 | SPRRARSVASQSII | WB |
| HLA-B*27:05 | RRARSVASQ | WB |
| HLA-B*27:05 | RRARSVASQSI | WB |
| HLA-B*27:05 | RRARSVASQSIIAY | WB |
| HLA-B*07:02 | RARSVASQSI | WB |
| HLA-B*27:05 | ARSVASQSI | WB |
| HLA-B*39:01 | ARSVASQSI | WB |
| HLA-B*27:05 | ARSVASQSIIAY | WB |
| HLA-B*58:01 | RSVASQSII | WB |
| HLA-A*01:01 | RSVASQSIIAY | WB |
| HLA-B*58:01 | RSVASQSIIAY | WB |
| HLA-B*15:01 | RSVASQSIIAY | WB |
| HLA-A*01:01 | SVASQSIIAY | WB |
| HLA-A*03:01 | SVASQSIIAY | WB |
| HLA-B*08:01 | SPRRARSV | SB |
| HLA-B*07:02 | SPRRARSVA | SB |
| HLA-B*07:02 | SPRRARSVASQSI | SB |
| HLA-A*26:01 | SVASQSIIAY | SB |
| HLA-B*15:01 | SVASQSIIAY | SB |
| HLA-B*07:02 | KPSKRSFI | WB |
| HLA-A*03:01 | SQILPDPSK | WB |
| HLA-B*39:01 | IKDFGGFNF | WB |
| HLA-A*03:01 | ILPDPSKPSK | SB |
| HLA-B*07:02 | DPSKPSKRSF | WB |
| HLA-B*27:05 | SKRSFIEDLL | WB |
| HLA-B*39:01 | SKRSFIEDLL | WB |
| HLA-B*07:02 | KPSKRSFIEDL | WB |
| HLA-B*07:02 | LPDPSKPSKRSF | WB |
| HLA-A*24:02 | ILPDPSKPSKRSF | WB |

**SUPPLEMENTARY TABLE 5: Predicted antigenic peptides spanning the SARS-CoV-2 S protein S1/S2 junction binding to MHC class 1 peptide binding using the NetMHC 4.0 server.**

Red= Peptides that overlap the furin cleavage site.

| **Peptide** | **MHC class 1 Alleles** |
| --- | --- |
| CDIPIGAGI | HLA-A1 HLA-A2 HLA-A*0201 HLA-A*0205 HLA-A*1101 HLA-A24 HLA-A3 HLA-A*3101 HLA-A*3302 HLA-A68.1 HLA-A20 Ca HLA-A2.1 HLA-B14 HLA-B*2702 HLA-B*2705 HLA-B*3501 HLA-B*3701 HLA-B*3801 HLA-B*3901 HLA-B*3902 HLA-B40 HLA-B*4403 HLA-B*5101 HLA-B*5102 HLA-B*5103 HLA-B*5201 HLA-B*5301 HLA-B*5401 HLA-B*51 HLA-B*5801 HLA-B60 HLA-B61 HLA-B62 HLA-B7 HLA-B*0702 HLA-B8 HLA-Cw*030 HLA-Cw*040 HLA-Cw*060 HLA-Cw*070 MHC-Db MHC-Db rev MHC-Dd MHC-Kb MHC-Kd MHC-Kk MHC-Ld |
| ECDIPIGAG | HLA-A1 HLA-A2 HLA-A*0201 HLA-A*0205 HLA-A*1101 HLA-A24 HLA-A3 HLA-A*3101 HLA-A*3302 HLA-A68.1 HLA-A20 Ca HLA-A2.1 HLA-B14 HLA-B*2702 HLA-B*2705 HLA-B*3501 HLA-B*3701 HLA-B*3801 HLA-B*3901 HLA-B*3902 HLA-B40 HLA-B*4403 HLA-B*5101 HLA-B*5102 HLA-B*5103 HLA-B*5201 HLA-B*5301 HLA-B*5401 HLA-B*51 HLA-B*5801 HLA-B60 HLA-B61 HLA-B62 HLA-B7 HLA-B*0702 HLA-B8 HLA-Cw*030 HLA-Cw*040 HLA-Cw*060 HLA-Cw*070 MHC-Db MHC-Db rev MHC-Dd MHC-Kb MHC-Kd MHC-Kk MHC-Ld |
| IGAGICASY | HLA-A1 HLA-A2 HLA-A*0201 HLA-A*0205 HLA-A*1101 HLA-A24 HLA-A3 HLA-A*3101 HLA-A*3302 HLA-A68.1 HLA-A20 Ca HLA-A2.1 HLA-B14 HLA-B*2702 HLA-B*2705 HLA-B*3501 HLA-B*3701 HLA-B*3801 HLA-B*3901 HLA-B*3902 HLA-B40 HLA-B*4403 HLA-B*5101 HLA-B*5102 HLA-B*5103 HLA-B*5201 HLA-B*5301 HLA-B*5401 HLA-B*51 HLA-B*5801 HLA-B60 HLA-B61 HLA-B62 HLA-B7 HLA-B*0702 HLA-B8 HLA-Cw*030 HLA-Cw*040 HLA-Cw*060 HLA-Cw*070 MHC-Db MHC-Db rev MHC-Dd MHC-Kb MHC-Kd MHC-Kk MHC-Ld |
| QTQTNSPRR | HLA-A1 HLA-A2 HLA-A*0201 HLA-A*0205 HLA-A*1101 HLA-A24 HLA-A3 HLA-A*3302 HLA-A68.1 HLA-A20 Ca HLA-A2.1 HLA-B14 HLA-B*2702 HLA-B*2705 HLA-B*3501 HLA-B*3701 HLA-B*3801 HLA-B*3901 HLA-B*3902 HLA-B40 HLA-B*4403 HLA-B*5101 HLA-B*5102 HLA-B*5103 HLA-B*5201 HLA-B*5301 HLA-B*5401 HLA-B*51 HLA-B*5801 HLA-B60 HLA-B61 HLA-B62 HLA-B7 HLA-B*0702 HLA-B8 HLA-Cw*030 HLA-Cw*040 HLA-Cw*060 HLA-Cw*070 MHC-Db rev MHC-Dd MHC-Kb MHC-Kd MHC-Kk MHC-Ld |
| CTSTMKIDL | HLA-A1HLA-A*1101HLA-B*3902HLA-B40HLA-B*5801HLA-B60HLA-B61MHC-KkMHC-Ld |
| DCMENTXML | HLA-A68.1HLA-B14HLA-B*3501HLA-B*3701HLA-B*3901HLA-B*4403HLA-B60HLA-B61HLA-B7HLA-B8HLA-Cw*0301MHC-DbMHC-Db MHC-Kd |
| EGDMVYGKL | HLA-A1HLA-A*3302HLA-B*3701HLA-B*3801HLA-B*3901HLA-B*4403HLA-B*5101HLA-B*5102HLA-B*5103HLA-B*5201HLA-Cw*0702MHC-DdMHC-KbMHC-Kk |
| HFIQNGVEL | HLA-A24HLA-B*3801HLA-B*0702HLA-Cw*0301HLA-Cw*0401MHC-DbMHC-Db MHC-Kd |
| IQNGVELRL | HLA-A2HLA-A*0205HLA-A24HLA-B*2702HLA-B*2705HLA-B*3701HLA-B*3801HLA-B*3902HLA-B*4403HLA-B*5201HLA-B60HLA-B62MHC-Kk |
| KNRELSREL | HLA-A24 HLA-B*3501HLA-B61HLA-B7HLA-B*0702HLA-B8MHC-Dd |
| LRNRFEAIL | HLA-B14 HLA-B*2702HLA-B*2705HLA-B*3902HLA-Cw*0702 |
| MLQCNAVNL | HLA-A2HLA-A*0201HLA-A*0205HLA-A20HLA-A2.1HLA-B*5301HLA-B*51HLA-B62MHC-DbMHC-Db MHC-DdMHC-KbMHC-Ld |
| MLVNDQEEL | HLA-A2HLA-A*0201HLA-A*3302HLA-A20HLA-A2.1HLA-B62MHC-DbMHC-Db MHC-KdMHC-Ld |
| MVYGKLMVL | HLA-A1HLA-A*0201HLA-A*0205HLA-A*1101HLA-A3HLA-A*3101HLA-A*3302HLA-A68.1HLA-B14HLA-B*2702HLA-B*2705HLA-B*3901HLA-B*5101HLA-B*5102HLA-B*5103HLA-B*5201HLA-B*5301HLA-B*5401HLA-B*51HLA-B7HLA-B8HLA-Cw*0301LA-Cw*0602MHC-DdMHC-Kb |
| PTHIASMFL | HLA-B*5801 |
| RVQLVLWID | HLA-A*1101HLA-A*3101 |
| SFMTMSKPL | HLA-A24HLA-B*3801HLA-B*3902HLA-B*5401HLA-B*0702HLA-Cw*0301HLA-Cw*0401MHC-KdMHC-Ld |
| TLKELILPL | HLA-A3HLA-A*3101HLA-A20HLA-A2.1HLA-B*3501HLA-B62HLA-B*0702HLA-B8HLA-Cw*0401LA-Cw*0602HLA-Cw*0702 |
| XLYTTLKEL | HLA-A2HLA-A*0201HLA-A*0205HLA-A3HLA-A20HLA-A2.1HLA-B14HLA-B*2702HLA-B*2705HLA-B*3901HLA-B*5101HLA-B*5102HLA-B*5201HLA-B*5301HLA-B*5401HLA-B*51HLA-Cw*0401HLA-Cw*0702MHC-Kb |
| YASLPNVFL | HLA-B*3501HLA-B*3701HLA-B40HLA-B*5101HLA-B*5102HLA-B*5103HLA-B*5301HLA-B*5401HLA-B*51HLA-B60HLA-B7 |

**SUPPLEMENTARY TABLE 6: Prediction of antigenic peptides spanning the SARS-CoV-2 S protein S1/S2 junction binding to MHC class 2 peptide binding by the NetMHCII server.**

Red= Peptides that overlap or comprise amino acids of the furin and/or TMPRSS2 cleavage sites.

| **Peptide** | | **MHC class 2 Alleles** |
| --- | --- | --- |
| IGAGICASY | HLA-DQA10301-DQB10301 HLA-DQA10501-DQB10303 HLA-DQA10301-DQB10301 HLA-DQA10501-DQB10303 HLA-DQA10501-DQB10301 HLA-DQA10501-DQB10301 | |
| QTNSPRRAR | HLA-DQA10201-DQB10402 HLA-DQA10303-DQB10402 HLA-DQA10201-DQB10402 HLA-DQA10303-DQB10402 HLA-DQA10201-DQB10402 HLA-DQA10303-DQB10402 HLA-DQA10201-DQB10402 HLA-DQA10303-DQB10402 HLA-DQA10303-DQB10402 HLA-DQA10303-DQB10402 HLA-DQA10201-DQB10402 HLA-DQA10201-DQB10402 HLA-DQA10201-DQB10402 HLA-DQA10303-DQB10402 HLA-DQA10201-DQB10402 HLA-DQA10303-DQB10402 HLA-DQA10201-DQB10402 HLA-DQA10303-DQB10402 HLA-DQA10201-DQB10402 HLA-DQA10303-DQB10402 HLA-DQA10303-DQB10402 HLA-DQA10303-DQB10402 HLA-DQA10201-DQB10402 HLA-DQA10201-DQB10402 | |
| RSVASQSII | H-2-IAs H-2-IAs HLA-DQA10103-DQB10603 HLA-DQA10201-DQB10402 HLA-DQA10103-DQB10603 HLA-DQA10201-DQB10402 H-2-IAs HLA-DQA10103-DQB10603 HLA-DQA10103-DQB10603 H-2-IAs HLA-DQA10103-DQB10603 HLA-DQA10103-DQB10603 H-2-IAs HLA-DPA10201-DPB11401 HLA-DPA10201-DPB11401 HLA-DQA10103-DQB10603 HLA-DQA10201-DQB10402 HLA-DPA10201-DPB11401 HLA-DQA10103-DQB10603 HLA-DQA10201-DQB10402 HLA-DQA10103-DQB10603 HLA-DQA10103-DQB10603 H-2-IAs HLA-DPA10201-DPB11401 HLA-DPA10201-DPB11401 HLA-DPA10201-DPB11401 | |
| SQSIIAYTM | | HLA-DQA10501-DQB10303 HLA-DQA10501-DQB10303 HLA-DQA10201-DQB10202 HLA-DQA10201-DQB10202 |
| VASQSIIAY | | DRB1_1201 DRB1_1201 DRB1_1201 DRB1_1201 DRB1_1201 DRB1_1201 DRB1_1201 DRB1_1201 |
| ILPDPSKPS | | DRB1_0301: DRB1_0305:  DRB1_0306: DRB1_0307: DRB1_0308: DRB1_0311: DRB1_0401: DRB1_0426: DRB1_1107: |
| FNFSQILPDPSKPS | | DRB1_0309: DRB1_0405: DRB1_0421: |
| FNFSQILPD | | DRB1_0801: DRB1_0817: DRB1_1101: DRB1_1128: DRB1_1305: DRB1_1321: DRB1_1502: |
| IKDFGGFNF | | DRB1_1501: DRB1_1506: |

**SUPPLEMENTARY TABLE 7: Predicted physiochemical properties of the polypeptides/protein subunits.** The lower the instability index II is, the more stable the protein is. The lower the GRAVY score is, the more soluble the protein is.

|  | **P3/FUR/x3** | **P4/TMP/x3** | **P3-L** | **SJ/FT** |
| --- | --- | --- | --- | --- |
| Number of amino acids | 63 | 42 | 107 | 230 |
| Molecular weight | 6183.61 | 3943.27 | 11105.12 | 24835.29 |
| Theoretical PI: | 12.90 | 10.64 | 10.55 | 4.85 |
| Number of negatively  (Asp + Glu) | 0 | 3 | 3 | 20 |
| Number of positively charged  (Arg + Lys) | 9 | 9 | 9 | 16 |
| Estimated half-life | - 30 hours (mammalian reticulocytes, *in vitro*) - >20 hours (yeast, *in vivo*). - >10 hours (*Escherichia coli, in vivo*)*.* | - 30 hours (mammalian reticulocytes, *in vitro*) - >20 hours (yeast, *in vivo*) - >10 hours (*Escherichia coli, in vivo*). | - 30 hours (mammalian reticulocytes, *in vitro*) - >20 hours (yeast, *in vivo*). - >10 hours (*Escherichia coli, in vivo*). | - 1 hour (mammalian reticulocytes, *in vitro*) - >30 min (yeast, *in vivo*). - >10 hours (*Escherichia coli, in vivo*). |
| Instability index (II) | 125.11 | 103.58 | 72.71 | 41.86 |
| Aliphatic index | 41.90 | 0.00 | 54.95 | 87.43 |
| Average of hydropathicity (GRAVY) | -0.933 | -1.757 | -0.535 | 0.050 |

**SUPPLEMENTARY TABLE 8: Immunization protocols of BALB/c mice using engineered polypeptides and protein subunits.**
